# *Contrast* – a lightweight Python framework for beamline orchestration and data acquisition

**DOI:** 10.1107/S1600577521005269

**Published:** 2021-06-08

**Authors:** Alexander Björling, Clemens Weninger, Maik Kahnt, Sebastian Kalbfleisch, Ulf Johansson, Simone Sala, Filip Lenrick, Karina Thånell

**Affiliations:** a MAX IV Laboratory, Lund University, Lund, Sweden; b Production and Materials Engineering, Lund University, Lund, Sweden

**Keywords:** data acquisition, instrument control, SCADA

## Abstract

The emergence of fourth-generation synchrotrons is prompting the development of new systems for experimental control and data acquisition. However, as general control systems are designed to cover a wide set of instruments and techniques, they tend to become large and complicated, at the cost of experimental flexibility. Here we present *Contrast*, a simple Python framework for interacting with beamline components, orchestrating experiments and managing data acquisition. The system is presented and demonstrated via its application at the NanoMAX beamline of the MAX IV Laboratory.

## Abstract 2

*Contrast*, a simple Python framework for controlling beamline experiments, is presented. *Contrast* can be easily adapted to any beamline, or serve as inspiration for constructing new, suitable tools tailored to individual instruments.

## 1. Introduction   

As next-generation light sources are being built around the world, the software systems that serve such machines are also developing. Incidentally, the development of these diffraction-limited storage rings has coincided with the rise of Python as the *de facto* standard for general-purpose scientific computing. As an interpreted language, Python natively provides scripting utilities and shells, which can be easily integrated in larger frameworks for interactive programmatic control. It is then not surprising that four new synchrotron data acquisition projects have been largely or entirely based on interactive Python. The Sardana (Coutinho *et al.*, 2011 ▸), bliss (Guijarro *et al.*, 2017 ▸) and Bluesky (Arkilic *et al.*, 2017 ▸) projects are written in Python, while GDA (Gibbons *et al.*, 2011 ▸) provides an embedded Jython interpreter.

The above-mentioned developments share a common purpose, namely to unify data acquisition procedures and pipelines. Here, bliss and GDA pursue this goal at the facility level, while the Sardana and Bluesky projects involve multiple laboratories. The advantage of this is that resources can be pooled to implement advanced features. For example, Bluesky provides very useful dynamic control, where experiments can be paused, rewound and resumed. Sardana allows building customized graphical interfaces using the *Taurus* and *Qt* toolkits, and provides generalized support for continuous scanning. While compelling, these high ambitions necessarily mean that code bases grow as wide ranges of functionality are added. At the time of writing, each of these projects contains well above 100000 lines of core source code. Clearly, generalizing and unifying data acquisition via large software projects can sometimes increase robustness and provide advanced functionality, but at a cost. As complexity grows, maintenance and code modification necessarily become more arduous.

On the other hand, experiments frequently demand high flexibility and the ability to adapt to unforeseen experimental needs. As an example of this, the NanoMAX beamline of the MAX IV Laboratory is a multi-modal instrument, where scanning and time-domain experiments can be designed by combining X-ray detectors and other equipment in flexible schemes. Experience from the beamline shows that the most productive acquisition system is one which the experimental scientist understands and is able to quickly modify, extend and troubleshoot in a transparent way. Indeed, we argue that the acquisition system is a core part of any beamline setup, which must be chosen or designed with the same care as taken when selecting or building instrument-specific hardware.

Conveniently, as large-scale research facilities typically run distributed control systems such as Tango (Chaize *et al.*, 1999 ▸) or EPICS (Dalesio *et al.*, 1994 ▸), the task of coordinating the various components of even the most complex experiment, and gathering the resulting data, can usually be broken down into rather simple operations. In this paper, we present a new and lightweight orchestration and acquisition framework, named *Contrast*, which provides the experimentalist with an interface for interacting with the underlying control system, as well as with a simple way to define experimental procedures (Björling, 2020*a* ▸). *Contrast* is deployed at NanoMAX, and has successfully been used for user experiments in scanning fluorescence spectroscopy, coherent imaging, tomography and *in situ* strain mapping for 18 months of user operation to date (Björling, 2020*b* ▸). Its deployment has brought increased flexibility, an improvement in beamline reliability, as well as the freedom to construct experimentally driven downstream analysis pipelines with relative ease.

While *Contrast* adds yet another Python-based acquisition framework, our ambition is not to provide a general system valid for any experimental context. We suggest that *Contrast* could be used as-is at other instruments, and that its simplicity would be an asset compared with the alternatives. But it can equally well serve as inspiration for how similar tailored systems can be built, showing by example that simple and transparent systems can substantially improve data acquisition.

The first part of the paper details the design choices and implementation. The second part describes its integration with the lower-level control system and detector infrastructure at NanoMAX. Lastly, we give two example applications of the framework, where X-ray fluorescence mapping and radial image sector integration are carried out and analyzed in real time as the data are collected.

## 2. Software design   

*Contrast* is a beamline interface based on IPython, with code organized as a library containing various classes. A beamline is set up simply by making instances of these classes for detectors, motors and any other devices directly in IPython or in a script.

*Contrast* conceptually does three things:

(i) Represents and keeps track of beamline components as Python objects.

(ii) Provides shorthand macros to carry out orchestrated operations on these devices.

(iii) Keeps track of the environment in which the instrument is run.

The framework runs locally in an IPython interpreter. This allows direct interaction between all beamline components. Parallelization is avoided in the interest of simplicity, with the exception of data handling, which would otherwise slow down the light-weight acquisition loop.

### 2.1. Object representation of beamline components   

**Figure 1:**
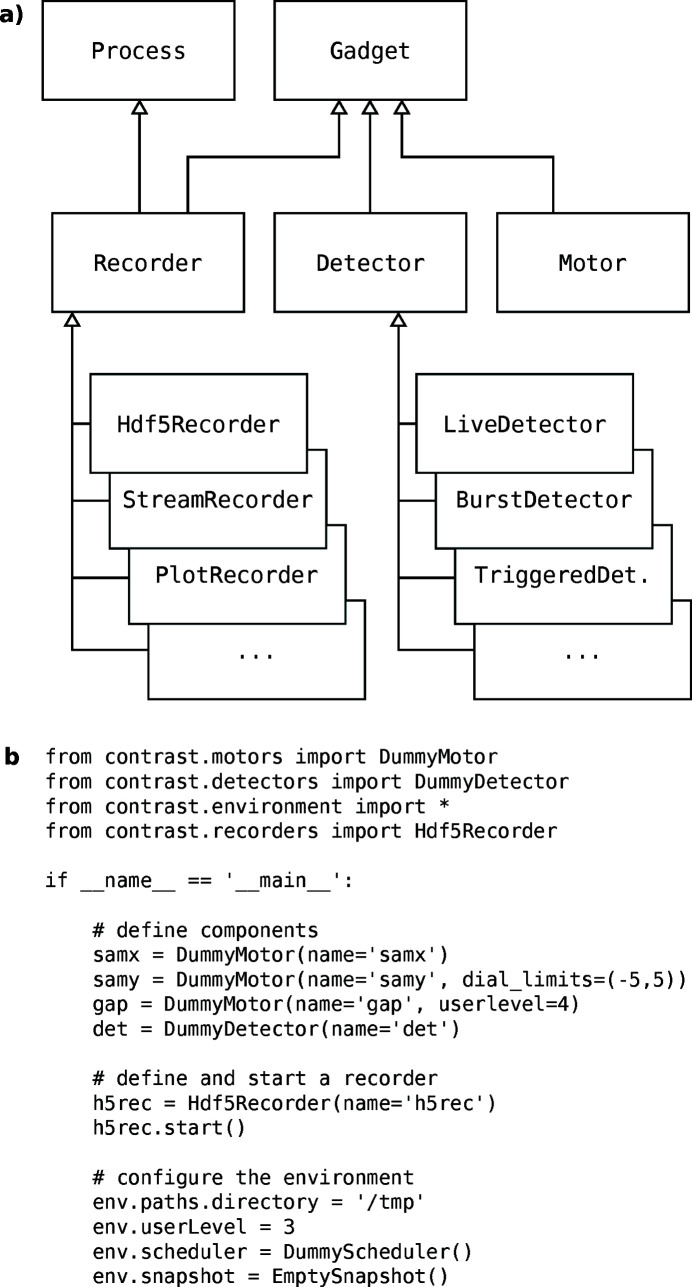
(*a*) Inheritance tree showing the most important beamline component base classes, along with three examples of 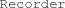 types. (*b*) Example script which launches a minimal dummy beamline. 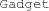 names are given as keyword arguments, so that the instance tracking can assign a unique name for each object.

Beamline components such as motors and detectors are represented by classes which expose a simple and uniform interface. At the top of the inheritance tree [Figure 1 ▸(*a*)], an abstract 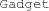 base class provides instance tracking, so that all objects can be found by interrogating class methods at the appropriate level in the tree. This allows querying all existing objects of a certain kind, without having to manage a global list of components.

The 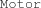 and 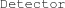 base classes represent beamline components that are primarily used for control and data acquisition, respectively. The distinction is not always clear, but devices are typically implemented as one or the other to satisfy one of the standardized interfaces. Actual motors, as well as voltage biases, temperature setpoints, sample pumps or collective coordinates of multiple real motors, for example, can usually conform to the 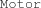 class interface. Data acquisition devices such as cameras, spectrometers, ion chambers, electrometers and other sensors can often conveniently inherit from 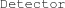 .

The 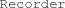 class is introduced in analogy with Sardana (Coutinho *et al.*, 2011 ▸) to handle incoming data asynchronously. Recorders are parallel sub-processes which perform tasks on collected data, for example relaying them over streams, writing them to file, or making live plots. Acquisition typically involves placing any gathered data in the queues of all currently active recorders (available through the 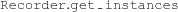 class method) before the measurement cycle continues without delay. High-rate detectors are assumed to pipe their data independently, with only links or other meta-information returned to *Contrast*.

Figure 1 ▸(*b*) shows a minimal beamline script, which sets up two motors, a detector and a data recorder. Such a script can be run interactively from within IPython using the 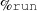 magic, or launched directly from a terminal as follows,

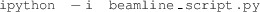

For a real instrument, the detector and motor objects created would correspond to the actual hardware, and instantiate hardware-specific subclasses of 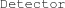 and 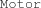 . Basic data handling would typically involve a simple 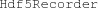 instance, while more elaborate schemes, as exemplified below, might make use of data distribution over *ZeroMQ* via instances of the built-in 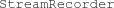 class. The documentation and source code (Björling, 2020*a* ▸,*b* ▸) contain detailed descriptions of all available components, as well as complete real-world examples.

Once the beamline is set up, the instance tracking by 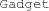 and its subclasses can be used to find any beamline component. Every 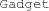 class tracks all of its instances, which can be accessed from anywhere in the library or from the interactive interpreter. For example, 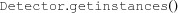 is a generator over all available detector objects, which can then be filtered on name, active status or some other property. Typically, however, shorthand macros, described in the next section, are used for handling components and running data acquisition procedures.

### 2.2. Shorthand macros   

**Figure 2:**
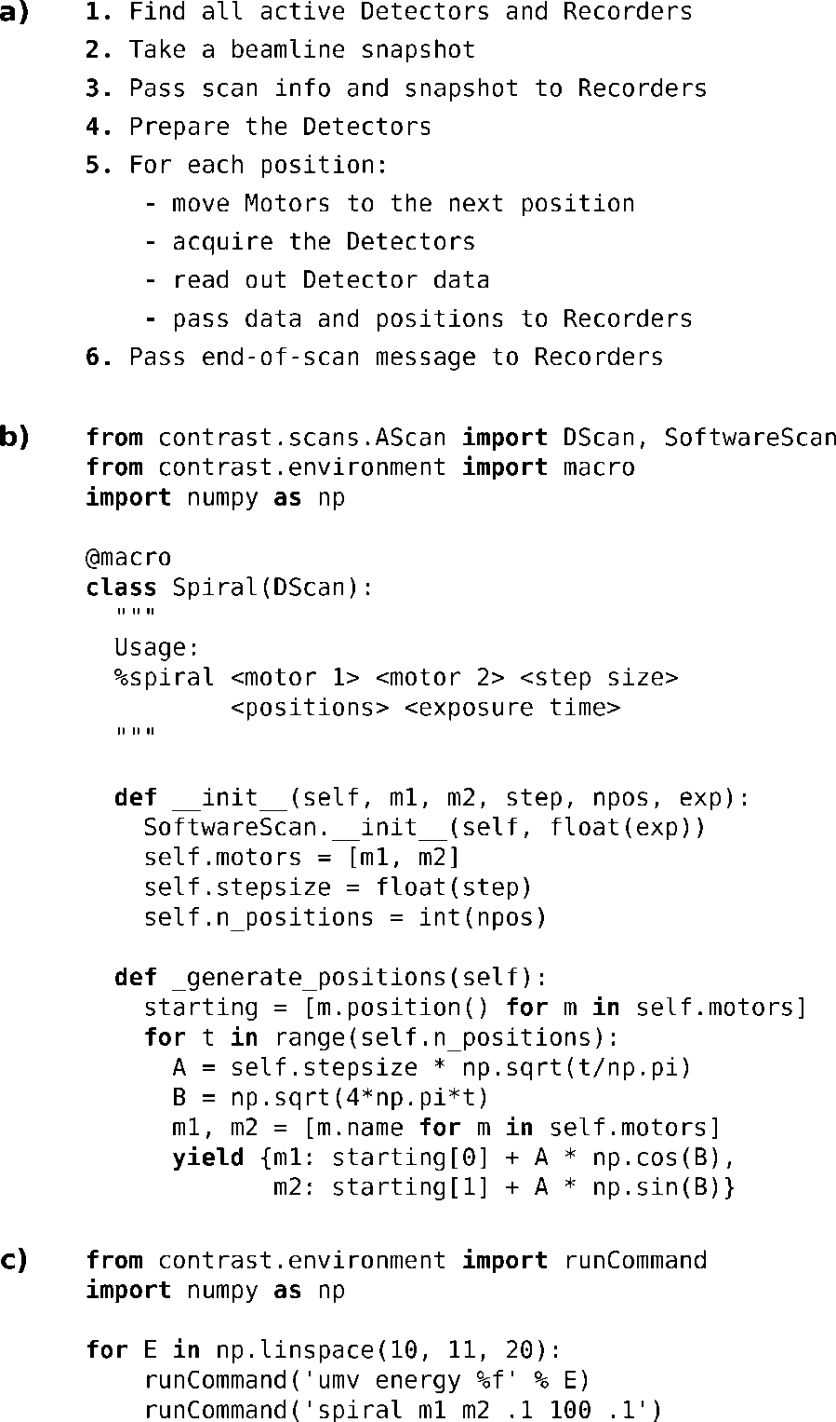
(*a*) Basic steps of a data acquisition macro. (*b*) Example code showing how to write a new macro, in this case implementing scanning of two motors along a spiral. (*c*) A simple procedural script in which a spiral scan is run across a range of *energy* motor positions.

The object representation of beamline components [Fig. 1 ▸(*a*)] already suggests how a basic acquisition loop might look. Typically, some motor or other parameter is varied. All active detectors are found through instance tracking, and then started at every parameter position. The data collected are then distributed to all available recorders for handling, before the cycle continues [see Figure 2 ▸(*a*)]. Any such sequences of operations on 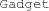 objects could be run directly on the IPython command line, or scripted in library functions. However, to provide a more user-friendly interface which also resembles familiar historical beamline acquisition systems, shorthand macros are introduced.

Macros are implemented as IPython ‘magic commands’, inspired by the interactive interpreter of Sardana (Coutinho *et al.*, 2011 ▸). This allows the user to enter short commands with shell-style arguments, rather than to require correct Python syntax. For example, the shorthand macro syntax

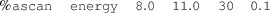

scans the *energy* motor from 8 to 11 keV in 30 intervals, exposing all active detectors for 0.1 s at each energy. This macro expression is equivalent to the following less convenient Python statement,

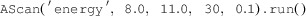

The *Contrast* library provides a decorator and class interface for writing custom macros. Macros are defined throughout the library source so that macros associated with certain classes are contained in the same module. For example, macros which operate on motors are found in the 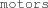 module, rather than being listed in a separate macro module, making the library intuitively organized and easy to version control. Macros can take optional keyword arguments, which is useful for enabling extra behaviour (*e.g.* adding positional jitter to mapping scans or adding extra delays). These do not have to be pre-defined, and can therefore be used for attaching arbitrary tags to experimental runs for later reference (*e.g.* to mark ptychographic scans for later automatic phase retrieval).

The built-in acquisition macros inherit from a common 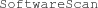 base class, which implements the core behaviour of actuating, measuring, collecting and redistributing data. The key steps of this sequence are illustrated as pseudo-code in Figure 2 ▸(*a*). Factoring out such core behaviour simplifies writing custom acquisition macros where only some part of the loop, for example in what pattern to scan some set of motors, is rewritten. Implementing new acquisition modalities can either build on the existing base classes, or start from scratch for entirely new functionality. For simple experimental procedures, ordinary Python scripts can be written to run a sequence of *Contrast* macros. Figure 2 ▸(*b*) shows how a two-dimensional spiral scan can be implemented as a proper *Contrast* macro, while Figure 2 ▸(*c*) illustrates a simple scripted procedure.

### 2.3. The beamline environment   

Aside from the core classes representing hardware components and data handlers, *Contrast* contains utility classes to manage and interface with other parts of the beamline. The 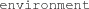 sub-module gathers these components as well as some macros to manage them. The sub-module creates a library-level object 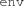 which can be globally imported. That way, data acquisition routines and 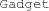 objects can interrogate the local environment if needed.

One important attribute on the central 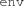 instance is the object which determines where to write data. By default, the attribute 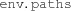 points to an object which takes data paths manually. But in real beamline environments, data paths might be set externally by the user management system, or be set from any other source. Another aspect of the environment is the conditions required for data acquisition to proceed. At synchrotrons, for example, conditions might be set on the storage ring current, electron injection status and state of the X-ray shutters. The 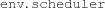 attribute can point to any customized class, and be interrogated by data acquisition routines.

Other beamline aspects managed by the central 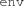 object include how to gather a beamline snapshot before acquiring data, a rudimentary user level setting for filtering the visibility of advanced beamline features, references to previous macro results, and an incremental scan number to identify acquired data. The last lines of Figure 1 ▸(*b*) illustrate how the 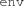 object can be configured to hold various utility objects. For an actual beamline, many of these will be adapted to the surrounding infrastructure. The actual 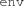 object for the NanoMAX instrument is described in Section 3 as a concrete example, and the documentation (Björling, 2020*b* ▸) can be consulted for a full description.

### 2.4. Comparison with existing frameworks   

A full, systematic comparison of data acquisition frameworks is beyond the scope of this article, especially as we have unbalanced experience of the above-mentioned frameworks. Clearly, *Contrast* has some disadvantages and lacks some attractive features compared with the heavier alternatives. As an example, beamline components are represented by objects within a Python process, and there is no standard way of interacting with them from the outside. Sardana, on the other hand, dynamically exposes a large set of Tango servers and builds on a server-client design. This allows running graphical user interfaces (GUIs) on different machines, and one could for example imagine implementing a web-based front-end for remote operation (even if, to our knowledge, this is not done). In *Contrast*, a GUI would instead be written as part of the main Python process, using *Qt* or some other widget toolkit. As another example, the Bluesky 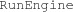 uses checkpointing for increased fault tolerance, which allows rewinding and re-running parts of experimental procedure. This feature is not available in *Contrast*, and there is no straightforward way of implementing such features. Also, convenience features such as the ability to manage queues of macros to run are not available in *Contrast*, where sequences are instead scripted as described above.

As outlined in the *Introduction* [Sec sec1], the development of *Contrast* was motivated by the need for a simpler acquisition framework. We define simplicity here in a practical sense, as describing a system which is easy for non-experts to understand and modify. As outlined above, this is necessary for a productive beamline as it allows experimentalists to adapt to changing needs. We consider *Contrast* simple in a number of ways, many of which we believe compare favourably with the existing alternatives.

First, the framework does not have a separate hardware abstraction layer, but instead the 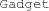 subclass representing a certain beamline component interfaces directly with the hardware or its server. As seen in the next section, this can be implemented in different ways depending on the hardware in question. For example, some detectors are fitted with full state machines with high-level interfaces, while others are managed by a separate control system (*e.g.* Tango) server via lower-level vendor libraries. When implementing a *Contrast* class to represent one of these components, these specifics are taken into account. This direct interface comes at the cost of potentially needing different 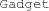 subclasses for the same hardware component, depending on how that component is run. On the other hand, it reduces the number of abstraction layers and makes it easier to follow the control flow and understand bugs and performance bottlenecks. This also means that the requirements on the other parts of the control system are very loose. Any interface can be tolerated, as long as the *Contrast* process is not required to perform heavy operations or time-consuming work. The current source code (Björling, 2020*a* ▸) contains examples of HTTP, PyTango and TCP socket interfaces, including blocking protocols which can be handled with local threads.

Secondly, *Contrast* strives to implement a clear basic API for the 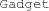 subclasses, so that the sequence in which components are moved, armed, triggered and read out is easy to follow. It is our experience that complicated control flows increase the risk of mistakes due to misunderstandings, especially when experimentalists who do not grasp the full framework design need to adapt to novel acquisition schemes, which can ultimately lead to unstable controls. In the simpler API design, all 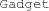 components are autonomous from the *Contrast* point of view. For example, a 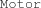 object defines a single degree of freedom, even if the motor itself is physically managed by a multi-axis motion controller. Where needed, managing classes which coordinate motions can be freely defined by the programmer, without the need for such meta-objects to be part of the *Contrast* framework. We note that, depending on the underlying hardware control, this could in principle mean that simultaneous or coordinated motions are not possible. However, with the motion controllers currently used at NanoMAX, this has not been encountered. The source code and documentation (Björling, 2020*b* ▸) contain several examples of multi-axis controllers. The reliance on the native Python import system in general allows the programmer to adapt to specific hardware, and to go beyond the scope of the thin API when needed.

Third, the *Contrast* design takes a minimal approach to error handling, with no ambition to comprehensively catch and handle exceptions. Since all 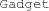 objects and data acquisition routines run in the same Python process, exceptions can be directly examined using built-in IPython debugger. By comparison, exceptions raised in the separate processes of more complex frameworks can often be hard to identify and understand. With a multitude of beamline components, for which the vendor-supplied servers or libraries can be of varying quality, the total set of possible errors, instabilities and deviations from specified behaviour is so wide that issues found are best dealt with on a per-component basis in the respective 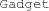 subclass. For general operations where network glitches or transient problems are found to impact operational stability, workarounds are implemented which for example try a number of times before re-raising the underlying exception.

We have found that the direct approach to hardware communication, the simple API and the transparent error handling greatly improve the operational beamline reliability. Thus the stability of the NanoMAX instrument, previously a bottleneck in user operation, was improved by deploying the simpler *Contrast* framework in place of the previous system.

## 3. The NanoMAX beamline   

We now turn to the application of *Contrast* to a real experimental system. NanoMAX is a general-purpose hard X-ray nanoprobe beamline at the MAX IV Laboratory, described in detail by Johansson *et al.* (2013 ▸) and Björling *et al.* (2020 ▸). While the current endstation is optimized for *in operando* diffraction experiments in the Bragg geometry, it also has forward imaging and X-ray fluorescence mapping capabilities. A second endstation for tomographic imaging is in the design phase. Hence, a wide set of detectors and other equipment is integrated via *Contrast*.

**Figure 3:**
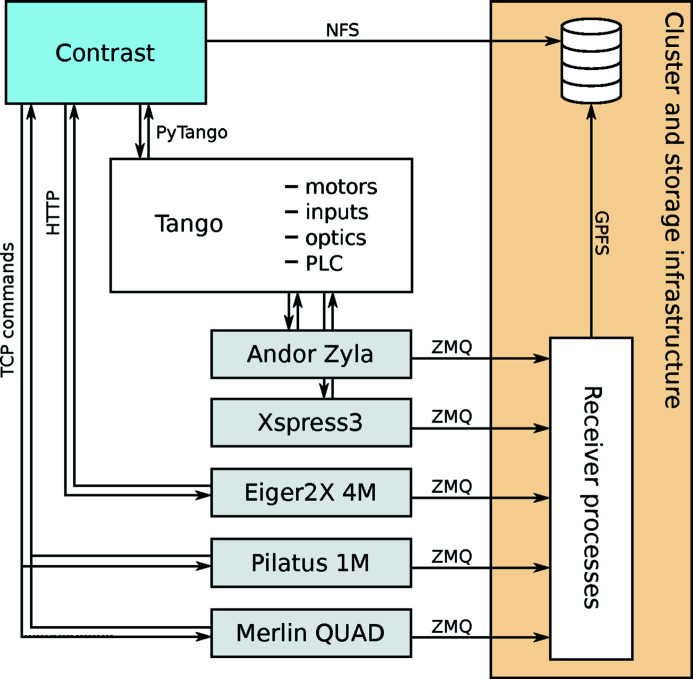
The NanoMAX data acquisition system in HDF5 saving mode. Tango is a distributed control system which manages all motors, interfaces with the beamline PLC and safety systems, controls the vacuum and optics, and handles data acquisition from encoders, electrometers, analog inputs, *etc*., as well as general sensor readout. These components are then orchestrated together with the high-rate detectors in *Contrast*. Note that Tango could be replaced by any distributed control system.

Figure 3 ▸ summarizes the NanoMAX acquisition system, including the main data pathways. The Merlin, Eiger and Pilatus detectors, for Bragg diffraction, forward imaging and wide-angle scattering, respectively, implement their own state machines and are interfaced directly through their representative *Contrast* objects. The Xspress3 and Andor detectors, for X-ray fluorescence emission spectroscopy (XRF) and forward full-field imaging, respectively, do not come with their own servers. For these two detectors, acquisition control is maintained by Tango servers built on vendor-supplied Software Development Kits (SDKs) (Björling, 2020*c* ▸; Weninger, 2020*a* ▸). All detectors stream data over *ZeroMQ* sockets directly to central cluster resources, where they are written to disk or analyzed in real time. For the Eiger, streaming is built into the vendor’s control server, while streaming solutions for the other detectors are written in-house (Weninger, 2020*b* ▸,*c* ▸). Figure 3 ▸ shows the basic mode of operation, where all detector data are simply written to disk as HDF5 files (The HDF Group, 2000–2020 ▸). Note that all high-rate detectors stream their data independently from *Contrast*. The built-in 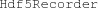 writes files in a simple, Nexus-inspired format, where consecutive motor values and detector data are appended to flat arrays. Streaming detectors return links to their separate target files, so that all data become accessible via the main *Contrast* HDF5 file. Since the 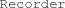 objects run asynchronously, low-rate data can be written directly over the Network File System (NFS) with no impact on the experimental overhead.

The beamline environment is taken into account by configuration of the central *Contrast* object 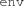 (Section 2.3). For example, data paths at MAX IV are managed by a central Scientific Data Management (SDM) system, which sets up storage folders based on user proposals. The data paths are available from a dedicated Tango device server, and the *Contrast* object 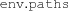 attribute refers to an instance of the 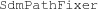 class, written to reflect this environment. This facility-specific object thus replaces the default 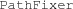 object, which would otherwise take paths manually. Similarly, the 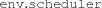 attribute refers to a 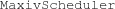 object which keeps track of the machine and safety shutter status at the beamline. This ensures that data acquisition can be paused when the beam is not available or is unstable due to electron injection. Following the API, the 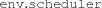 object can also report on upcoming deadlines, such as injections, and macros are free to estimate whether there is enough time for another acquisition before the next such event. The other 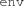 attributes used at NanoMAX follow the library defaults.

Data collection at NanoMAX frequently involves scanning a sample through the beam, for example in mapping or ptychographic imaging. While such scans can be synchronized via *Contrast*, it is often more efficient to delegate motion control and triggering to designated hardware. This also allows performing continuous scanning to avoid the overhead of the scanner’s settling time. At NanoMAX, delegation of scanning control to an nPoint three-axis piezoelectric stage is implemented as a dedicated macro. For each software step, the macro parameters are used to calculate the desired piezo motor waveform. Any detector which can be hardware triggered is then armed for the appropriate number of exposures, while non-triggered sensors are set up for a single point of data acquisition. As the waveform starts, the motion controller sends a signal to a PandABox acquisition board (Zhang *et al.*, 2017 ▸), which produces a time-based trigger train for the detectors, and also reads the piezo encoder signals in a gated-average fashion. After waveform execution, control is returned to *Contrast*, which passes the gathered data to recorders as usual, and proceeds to the next software step.

## 4. Example applications with real-time analysis   

Beyond the basic operating mode described in Figure 3 ▸, *Contrast* is used at NanoMAX together with streaming detectors to form real-time data analysis pipelines as needed. These online analysis schemes extend the basic HDF5 writing scheme in Figure 3 ▸ in ways particular to specific experimental applications. While not part of *Contrast* itself, these examples are included to demonstrate that advanced data pipelines can be built from simple elements in combination with *Contrast*’s 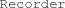 objects.

### 4.1. XRF mapping   

**Figure 4:**
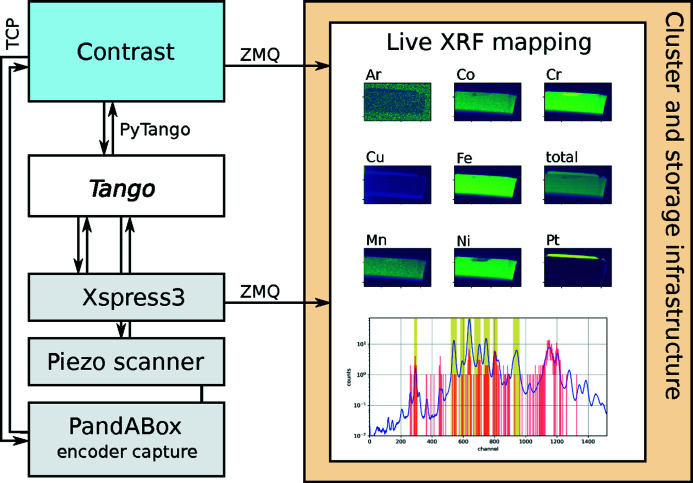
Real-time XRF mapping using *Contrast* and streaming detectors at NanoMAX. Selected parts of the beamline are shown schematically. The live processing pipeline plots the most recent fluorescence emission spectrum (red), along with the average of the spectra received (blue). Also shown in yellow bands are the selected spectral regions of interest. Maps are created for each such region, here corresponding to eight elements and the total spectrum. The decrease in Ar signal across the lamella comes from shadowing the air path behind the sample.

The first example is illustrated in Figure 4 ▸, where continuous scanning and XRF detection are combined with a stream receiver which creates and updates elemental maps as the data become available. Here, the nPoint LC.400 device controlling the piezo scanner generates continuous scanning patterns, and XRF data are collected from the moving sample, as described above. The positional data are streamed out by a *Contrast* 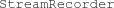 , while the Xspress3 pulse processor is managed by a Tango server with streaming capabilities (Björling, 2020*c* ▸).

The measured sample is a lamella of heat-treated steel, prepared by focused ion beam (FIB) milling. The lamella is approximately 10 µm high. It contains several first-row transition metals, as well as Pt from the FIB preparation. Before the scan, a selection is made of spectral ranges corresponding to each element of interest, as determined from a preliminary scan. This reduces the memory usage of the application, since each spectrum can be reduced to set of scalars as it is received. As the scan progresses, the XRF map for each element develops and grows, while the latest and average emission spectra are updated for the user to follow the experimental progress.

The example shown here reveals differences in elemental distribution. While Fe, Co and Mn are evenly spread across the lamella, there is complementary structure to Ni and Cr. Having this type of information available during data collection allows beamline users to make informed decisions on the further progress of the experiment, cancel scans after passing the region of interest, and gain a general sense of how the data acquisition is going. A video of this data collection and live processing is shown in the supporting information online. The supporting information also contains a video showing the live processing of an XRF scan across a collection of gold-capped gallium phosphide nanowires grown by gas-phase epitaxy (Sivakumar *et al.*, 2020 ▸).

This live mapping application was possible to realize with relative ease, thanks to the built-in 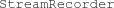 which gives access to all data collected by *Contrast* anywhere on the local network. Additionally, using streaming detectors allows for conveniently combining high-rate data with that from *Contrast* in custom downstream analysis or feedback utilities. Another application, not yet implemented, could be real-time phase retrieval of coherent imaging data, for example live ptychographic reconstruction (Daurer *et al.*, 2017 ▸).

### 4.2. Live radial integration   

Another example of real-time analysis is the radial integration of X-ray scattering (SAXS/WAXS) or powder diffraction (XRD) data. Experiments in scanning SAXS/WAXS or XRD often use exposure times on the order of 10 ms, with a resulting frame rate of 100 Hz. For time-resolved or time-correlation techniques, the Eiger2X 4M installed at NanoMAX is often operated up to its maximum frame rate of 500 Hz, corresponding to a raw data rate of up to 36 Gbit s^−1^. In order to obtain fast experimental feedback on the experiment, it is important to be able to radially integrate this deluge of image data in rings or sectors without significant delay.

**Figure 5:**
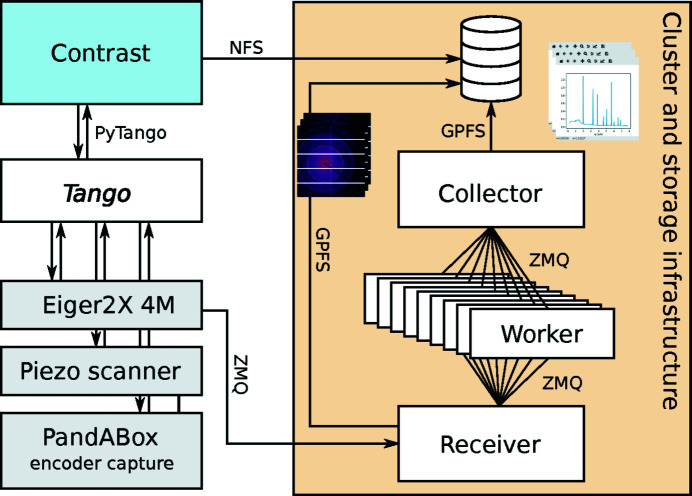
Real-time radial integration of Eiger 4M images at 500 Hz. The rate test used 12 worker processes. All HDF5 writers create separate files, which are linked from the main *Contrast* HDF5 file for single-point access.

Figure 5 ▸ shows a schematic layout of an acquisition setup where 4M Eiger images are integrated as they are collected (Weninger, 2020*d* ▸). The primary receiver of the detector stream writes everything to disk and relays the compressed data on a secondary *ZeroMQ* socket. The images are then distributed to a set of worker processes by a dynamic round-robin schema through the *ZeroMQ* push/pull pattern. The workers are dedicated to radial integration using a compiled library module for fast processing. A collector process gathers and orders the integrated data, before writing to disk in the HDF5 format. As soon as the acquisition is complete, the file is closed and the integrated data are available for inspection. Tests at NanoMAX show that the pipeline can keep up with the maximum frame rate of the Eiger2X 4M of 500 Hz using 12 worker processes on a compute node with two 12-core CPUs (Intel Xeon Gold 5118).

The scheme with a secondary processing pipeline is also utilized for other intra-frame operations such as down-sampling, with similar performance. Additional tasks such as frame-by-frame hit finding could be readily added to the existing pipeline. Inter-frame analyses based on reduced data, such as beam damage assessment or live calculation of time–time correlations, could be run in the collecting process to suit the needs of the particular experiment, but are not currently implemented.

## 5. Conclusion   

We have presented the basic design of the *Contrast* acquisition system, as well as its implementation at the NanoMAX beamline with two examples of real-time analysis pipelines. Although *Contrast* could be installed and used at other instruments with relative ease, it also serves as an example of how simple beamline-driven acquisition systems can improve the performance of an instrument. We argue that experimental control is at the heart of modern beamlines, and should be treated on equal footing with other scientific tools.

Clearly, the cost of developing and maintaining the software must also be factored into strategic beamline controls decisions. Here, taking part in broader software collaborations can ideally reduce the work needed per beamline for implementing experimental control. Also, for large facilities, standardization allows pooling of resources and enables central support and responsibility for the software. But as discussed above, experience from the particular case of the NanoMAX beamline shows that these costs were outweighed by the advantages.

## Supplementary Material

An animation of the live XRF mapping experiment from Figure 4. DOI: 10.1107/S1600577521005269/fv5135sup1.gif

Animated live processing of an XRF mapping scan across a collection of gold-capped gallium phosphide nanowires. DOI: 10.1107/S1600577521005269/fv5135sup2.gif
