# Supplementary figures and images for: Contrast – a lightweight Python framework for beamline orchestration and data acquisition
Source: J Synchrotron Radiat. 2021 Jun 8;28(Pt 4):1253–60. doi: 10.1107/S1600577521005269 (PMC8284407; doi:10.1107/S1600577521005269)

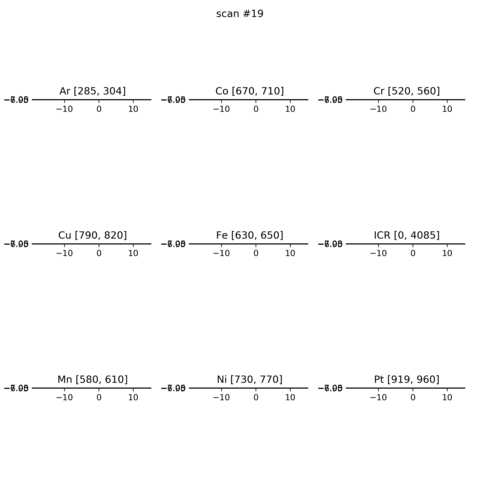

Supplement: Supplementary file 1 [file s-28-01253-sup1.gif]

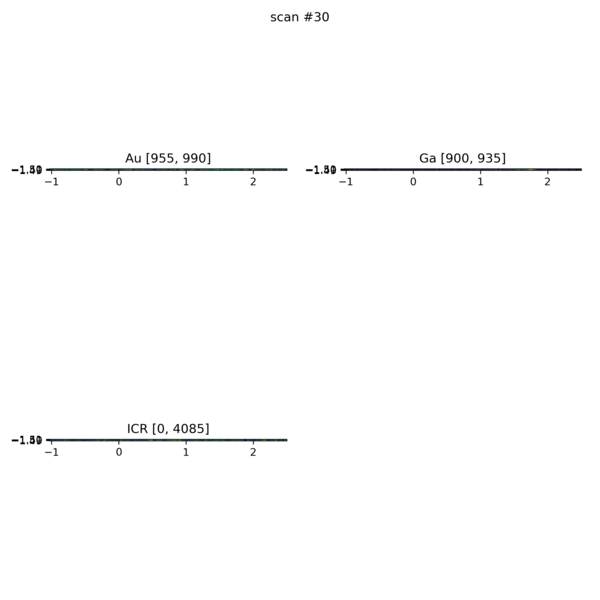

Supplement: Supplementary file 2 [file s-28-01253-sup2.gif]
